# Treatment of Rheumatism by Inoculation with Morphia

**Published:** 1841-09

**Authors:** Q. Gibbon


*Treatment of Rheumatism by Inoculation with Morphia.* By Q. Gibbon, M. D.

Salem, N. J., May 19th, 1841.

*To Professor Dunglison.*

Dear Sir,—A wish to present to the profession what I deem a useful fact, has iuduced me, though at the risk of intruding upon the time of one with whom I have not had the pleasure of a personal acquaintance, to offer for your consideration the following remarks:—

Having seen in your work upon "New Remedies," published sometime since in the "Library," an article upon the beneficial effects derived from inoculation with morphia in the treatment of local diseases, I determined to make a trial of the plan in local rheumatism, an affection which I have frequently found great difficulty in managing. I was not long afterward called to treat an obstinate rheumatic affection of the knee-joint, which had resisted the usual general and local means. One quarter of a grain of the sulphate of morphia was inserted, by means of punctures, in the skin over the affected part, twice a day for two days, with the most marked and satisfactory results. The patient, who for two weeks previous had suffered extreme pain upon the slightest motion of the limb, was, at the expiration of this period, able to walk with slight inconvenience, and upon the third day, threw aside the crutch with which he had before hobbled across his room. Friction with stramonium ointment, two or three times repeated during the fourth day, removed all remaining disease in the affected part.

The next case was a rheumatism of the wrist, in which no previous treatment had been practised. In this case, which was recent, two applications of the morphia, upon two succeeding days, so effectually removed the pain and tenderness, as to allow of the free use of the hand on the third day.

The third trial was made upon an obstinate rheumatism of the knee-joint, remaining after the subsidence of general rheumatism. The patient, a boy of sixteen, after having been rendered motionless for several days by a severe attack of inflammatory rheumatism of the whole system, recovered under the free use of tart. antim. opium, and colchicum, with the exception of the knee in question, which remained exquisitely painful, and tender upon pressure in one spot of about an inch in diameter upon its inner side. The insertion of a quarter of a grain of morphia, produced in a few hours a decided impression upon the pain, and by the second day, the symptoms were so far mitigated as to permit free motion in the part. This patient recovered rapidly without any farther medication.

A fourth case, which had resisted acupuncturation, assisted by the free use of the stramonium ointment, yielded upon the fourth day from the first application of the morphia, the patient expressing much satisfaction at the effects of the remedy.

Since treating the above cases, I have had an opportunity of testing the good effects of morphia in several other instances, and with results which induce me to entertain a very favorable opinion of its remedial powers. Judging from the limited experience which I have as yet had of its application, I should think it best adapted to the recent and active grades of the disease. I think it proper, however, to state that I have met with one case of rheumatism in which, though to all appearance a favorable one for its successful ehibition, the morphia failed in producing its beneficial effects.—*American Medical Intelligencer.*

